# Phenytoin Augmentation of Levetiracetam Treatment: A Case of Forced Normalization With Emergence of Psychosis

**DOI:** 10.7759/cureus.2432

**Published:** 2018-04-05

**Authors:** Michael Esang, Vijaya Padma Kotapati, Saeed Ahmed

**Affiliations:** 1 Behavioral Health Sciences, Nassau University Medical Center, East Meadow, USA; 2 Psychiatry, Manhattan Psychiatric Center

**Keywords:** forced normalization, psychoses, kindling, epilepsy seizures, seizure disorder, seizure control convulsions., levetiracetam, phenytoin, anticonvulsant, antiepileptic (aed)-induced psychotic disorder (aipd)

## Abstract

Forced normalization is the emergence of psychoses following stabilization of seizures in an uncontrolled epileptic patient. The current study is a case of forced normalization, a phenomenon characterized by normalization of electroencephalogram (EEG) findings and resolution of seizures. This case report is unique and rare because the patient meets the diagnostic merit of forced normalization, which occurred due to a nonconventional method of seizure control management. We discuss the recognition and differential diagnoses of such cases, understanding the phenomenon of forced normalization and treatment strategies, which may help clinicians in their clinical practice.

## Introduction

The presentations of epilepsy and psychiatric conditions could be complicated and challenging to diagnose and treat because these conditions are often interrelated. There is a higher risk of psychosis in patients with epilepsy up to 6-12 times greater than the general population. The literature also shows cases of psychosis following the use of anti-epileptic drugs (AEDs) such as phenytoin and levetiracetam in epileptic patients [1-2]. Not only can each AED cause psychosis, but a mixed-methods study by Chen Z et al., (2016) revealed that the concurrent use of levetiracetam with another AED poses a serious risk of developing an anti-epileptic-induced psychosis in patients with epilepsy [2]. Although research has shown that levetiracetam is well-tolerated with a favorable pharmacokinetic profile, neuropsychiatric adverse effects have been reported in some levetiracetam users, especially those with pre-existing psychiatric disorders, rapid titration, and those on add-on therapy [3-5]. Epileptic psychoses are also associated with the phenomenon called forced normalization process, which is characterized by an acute or sub-acute onset of psychosis associated with a dramatic reduction of epileptiform activity [5].

## Case presentation

A 26-year-old female with a history of a seizure disorder and no past psychiatric history presented to the emergency room (ER) in an altered mental state (AMS), exhibiting psychotic symptoms, all acute onset. She was admitted to the inpatient medicine unit, where she was co-managed by consultation-liaison psychiatry and neurology services. Her family revealed that she was diagnosed with the seizure disorder in late 2016 and had been treated with levetiracetam 500 mg twice a day (BID) until October 2017. However, in November 2017, she began to have severe and frequent seizure episodes. Due to increased severity and frequency of these episodes, her private neurologist added phenytoin 100 mg three times a day (TID) instead of titrating levetiracetam 500 mg BID. According to her family, soon after she began taking phenytoin, she became more irritable, labile, and was in "rage all day long”, which eventually escalated into aggression, agitation, and florid psychosis, and led to the hospitalization. During her stay on the medical floor, therapeutic levels of phenytoin and levetiracetam were 18 μg/mL (normal range: 10.0-20.0 μg/mL) and 11.2 μg/mL (normal range: 12-46 μg/mL), respectively. The patient’s electroencephalogram (EEG) was normal, her computed tomography (CT) head scan was unremarkable, and her pregnancy test, antinuclear antibody (ANA), and Lyme disease serologies were negative. Her erythrocyte sedimentation rate (ESR), folate, and vitamin B12 levels were also within normal limits.

During her hospitalization, the severe agitation was treated with Haldol 5 mg and lorazepam 2 mg, both administered intramuscularly every 8 hours as needed. Levetiracetam was discontinued, and the patient was started on divalproex sodium ER 500 mg BD. Intravenous fluid administration addressed the patient's elevated levels of creatinine kinase (1088 I/L to 3061 U/L) until they dropped to 420 U/L. When her aggression resolved, she was started on oral olanzapine 2.5 mg to address psychosis. Subsequently, the patient's condition stabilized, and she was discharged. She received follow-up care at the adult medicine, psychiatry, and neurology clinics. At the time of discharge, her medications were phenytoin 100 mg TID, olanzapine 2.5 mg BID, and divalproex sodium ER 500 mg BID.

## Discussion

Forced normalization occurs with the emergence of psychosis following an effort to control seizures, regardless of the control method used, e.g., medication, surgery, or neurostimulation. The kindling induced by electric or neurotransmitter processes is thought to result in behavioral changes.

This case emphasizes the importance of appropriate selection of anti-epileptics and closely monitoring epileptic patients who are at increased risk for neuropsychiatric complications [6]. As we know, patients with epilepsy have a higher risk of psychotic disorders [7-8]. Previously published literature suggests that levetiracetam is one of the most common AEDs taken by patients with an AED-induced psychotic disorder (AIPD); 57.1% of the study’s participants took levetiracetam, either alone or in combination with other drugs [2]. Levetiracetam causes irritability and aggression to a certain level; however, more serious concern arises when a patient with epilepsy presents psychotic symptoms with the use of levetiracetam [2].

This case highlights the important clinical aspect that many clinicians may encounter in their daily practice when a patient is already on one AED such as levetiracetam and continues to have seizure episodes. So prescribers are often inclined to titrate the medication or add another AED in the ongoing regimen. This is a point where forced normalization occurs because of continuous efforts to control seizures, which may lead to psychosis. As we noticed in this case, the phenomenon of the forced normalization process disappeared when levetiracetam was withdrawn, which provides clinical evidence to support the hypothesis that adding a second AED on top of ongoing AED regimen can cause forced normalization. We understand that maintaining a balance between adequate seizure control and the patient’s ability to function without overt psychopathology is crucial, but some of our patients may develop neuropsychiatric complications that warrant close monitoring to prevent grave consequences such as psychosis. We hypothesize that neuropsychiatric complication such as psychosis could occur not only because of rapid titration medication i.e., LVT but by adding an AED on top of an AED such as levetiracetam as described in the above case.

## Conclusions

Because certain AEDs have a greater tendency to induce psychosis, divalproex sodium ER was substituted for levetiracetam in this case. Another possible substitute would be carbamazepine, as this drug carries a low risk of psychotic side effects and has instead been shown to reduce aggression, anxiety, and manic effects and mood stabilizer properties. We recommend that providers consider these alternate options instead of using a conventional treatment, which may place these patients at risk of developing neuropsychiatric complications. In the management of AIPD, olanzapine should be considered because of its advantages over other antipsychotics (except clozapine). Olanzapine has anticonvulsant properties, which can be attributed to the increased release of allopregnanolone, an endogenous neuroactive steroid. For this reason, the indexed patient was stabilized with olanzapine due to its compelling anticonvulsant benefits. The patient was discharged with outpatient follow up at our outpatient clinic and has had no occurrence of neuropsychiatric symptoms or seizures until the date of reporting this case study.
